# NLRP3 Inflammasome: A Possible Link Between Obesity-Associated Low-Grade Chronic Inflammation and Colorectal Cancer Development

**DOI:** 10.3389/fimmu.2018.02918

**Published:** 2018-12-11

**Authors:** Patricia Ahechu, Gabriel Zozaya, Pablo Martí, José Luis Hernández-Lizoáin, Jorge Baixauli, Xabier Unamuno, Gema Frühbeck, Victoria Catalán

**Affiliations:** ^1^Department of Surgery, Clínica Universidad de Navarra, Pamplona, Spain; ^2^Metabolic Research Laboratory, Clínica Universidad de Navarra, Pamplona, Spain; ^3^CIBER Fisiopatología de la Obesidad y Nutrición, Instituto de Salud Carlos III, Pamplona, Spain; ^4^Obesity and Adipobiology Group, Instituto de Investigación Sanitaria de Navarra, Pamplona, Spain; ^5^Department of Endocrinology & Nutrition, Clínica Universidad de Navarra, Pamplona, Spain

**Keywords:** inflammasome, NLRP3, inflammation, colon cancer, obesity, adipose tissue, immunity

## Abstract

Emerging evidence reveals that adipose tissue-associated inflammation is a main mechanism whereby obesity promotes colorectal cancer risk and progression. Increased inflammasome activity in adipose tissue has been proposed as an important mediator of obesity-induced inflammation and insulin resistance development. Chronic inflammation in tumor microenvironments has a great impact on tumor development and immunity, representing a key factor in the response to therapy. In this context, the inflammasomes, main components of the innate immune system, play an important role in cancer development showing tumor promoting or tumor suppressive actions depending on the type of tumor, the specific inflammasome involved, and the downstream effector molecules. The inflammasomes are large multiprotein complexes with the capacity to regulate the activation of caspase-1. In turn, caspase-1 enhances the proteolytic cleavage and the secretion of the inflammatory cytokines interleukin (IL)-1β and IL-18, leading to infiltration of more immune cells and resulting in the generation and maintenance of an inflammatory microenvironment surrounding cancer cells. The inflammasomes also regulate pyroptosis, a rapid and inflammation-associated form of cell death. Recent studies indicate that the inflammasomes can be activated by fatty acids and high glucose levels linking metabolic danger signals to the activation of inflammation and cancer development. These data suggest that activation of the inflammasomes may represent a crucial step in the obesity-associated cancer development. This review will also focus on the potential of inflammasome-activated pathways to develop new therapeutic strategies for the prevention and treatment of obesity-associated colorectal cancer development.

## Introduction

During the past decades, colorectal cancer (CRC) has become a major public health problem constituting the second most common type of cancer in females and the third most common in males, with its incidence being expected to continue increasing in the coming years (1, 2). Colorectal carcinogenesis is a heterogeneous process characterized by different sets of molecular alterations influenced by gender, diet, environmental and microbial exposures as well as host immunity (3). Recently, global epidemiological and scientific studies suggest that obesity, a modifiable lifestyle factor, influences not only the risk of CRC incidence but also its morbidity and mortality (4–7). Different meta-analyses have revealed that an increased body mass index (BMI) is related with an elevated incidence of CRC (8–10) and with each unit increase of the BMI, the risk for CRC augments by 2–3% (11–13). Additionally, patients with type 2 diabetes (T2D) also have an increased risk for CRC development (14–16). These data are alarming because globally, the prevalence of obesity is reaching epidemic proportions worldwide (17–19). It is crucial to distinguish between the different anatomical distribution of the fat depots, rather than focusing exclusively on the BMI, given that abdominal obesity characterized by an accumulation of visceral fat is associated with metabolic risk whereas the peripheral subcutaneous fat depot confers protective effects on energy homeostasis (20, 21). In this line, cancer risk has been shown to be diminished among metabolically healthy overweight/obese adults as compared to overweight/obese adults with metabolic dysfunction (22).

Although mechanistic insights explaining these data are notably lacking, three main factors are considered to link excess body fat and CRC: (i) alterations in the metabolism of endogenous sex steroids, (ii) the insulin-insulin like growth factor (IGF)-1 axis, and (iii) the adipocyte-derived cytokines (named adipokines) (6, 23–25). Importantly, obesity is defined not only by an excess of adipose tissue accumulation but also by an altered adipose biology and all these previous factors are closely connected to the typical dysfunction of adipose tissue in the obese state (26). It is widely accepted that adipose tissue from obese individuals exhibits a sustained chronic and unresolved inflammation (27, 28) that is associated with an imbalance of adaptive homeostatic mechanisms and leads to the development of obesity-associated comorbitidites (29). Thus, growing evidence indicates that obesity-associated low grade chronic inflammation is a central mechanism whereby obesity promotes CRC risk and progression (30, 31). In this sense, multiple clinical studies have evaluated the role of inflammation as a possible mechanism linking adipose tissue excess with increased risk of CRC development (6).

## Inflammation, Obesity, and Colorectal Cancer

In 1863, Rudolf Virchow described the localization of leukocytes in neoplastic tissues suggesting a connection between inflammation and cancer. He proposed that the “lymphoreticular infiltrate” indicated the induction of carcinogenesis at sites of chronic inflammation (32). Much of our understanding of the close link between chronic inflammation and the development of CRC is illustrated by elegant studies showing that in patients suffering from chronic inflammatory bowel disease of the colon, the incidence of CRC increases progressively over time, reaching 19% after 30 years of disease (33–36) Furthermore, population-based studies have demonstrated an increased risk of cancer in individuals who are predisposed to develop specific types of chronic inflammatory diseases (37). Significantly, obesity is associated with chronic inflammation, both systemic and at the tissue level (38). Extensive experimentation has demonstrated that immune cells, including lymphocytes and macrophages, infiltrated visceral adipose tissue from patients with obesity inducing a complex immune response (38, 39). Indeed, adipose tissue macrophages (ATM) exhibit plasticity, changing their phenotype and functions in response to both the surrounding microenvironment and external stimuli (29). ATM in healthy fat depots generally exhibit a dominant M2 phenotype but during obesity, a polarization toward a pro-inflammatory M1 macrophage profile has been evidenced (39, 40). Pro-inflammatory ATM produce tumor-promoting cytokines, including tumor necrosis factor (TNF)-α, interleukin (IL)-1β, IL-6, IL-8, IL-18, IL-32, interferon (IFN)-γ, vascular endothelial growth factor (VEGF), osteopontin (OPN), tenascin C (TNC), and monocyte chemoattractant protein (MCP)-1 (24, 41). Thereby, adipose tissue from patients with obesity becomes a chronically impaired tissue constituting an important source of proinflammatory mediators, potentially fostering tumor growth (30). In addition, increased circulating levels of pro-inflammatory cytokines are typically found in obese patients, promoting a microenvironment favorable for tumor growth (42–47). Unresolved inflammation favors a complex paracrine signaling mechanism between innate immune cells and cancer cells (48, 49). Studies performed in conditional-knockout mice treated with specific carcinogenenic compounds to promote inflammation-induced colitis-associated cancer, have shown that the inactivation of the nuclear factor κ B (NF-κB) signaling in tumor infiltrating inflammatory cells results in attenuated colon cancer formation (49). Therefore, one conceptual issue to define is that many of the immune cells subsets and molecules that control inflammation influence immune reactions, and vice versa (31).

Besides chronic inflammation, critically involved in tumor progression, experimental and clinical evidence has unveiled a protective role for immune cells in cancer progression (50). This evidence led to the hypothesis of a duality of the innate immune system in tumor evolution exhibiting both oncogenic and tumor suppressive properties (51). Specifically, inflammasomes, as major mediators of the innate immunity, also show a dual role in inflammation contributing to carcinogenesis or immunosurveillance in a highly context-dependent fashion (31, 52). This ambivalent contribution is most probably due to the specific activities and properties of the multiple inflammasome complexes that are dependent on the simultaneous expression of their specific constituents in the same cell type of damaged tissues (53).

## Role of Hypoxia-Associated Inflammation in Obesity And Colorectal Cancer

Maintenance of oxygen homeostasis is essential for cellular and tissue function. Hypoxia results from a constant inadequate supply of oxygen (O_2_), resulting in decreased O_2_ tension that limits its availability to the demands of the surrounding tissue (54). Hypoxic stress is clinically relevant and plays a pivotal role in the pathogenesis of multiple human diseases, including cardiovascular diseases, diabetes, and cancer (55). In the obese state, adipose tissue depots become hypoxic as tissue mass expands, restricting the availability of oxygen particularly in clusters of adipocytes that are distant from the vasculature (56). This imbalance between the O_2_ supply and demand in adipocytes promotes the release of pro-inflammatory mediators, an inhibition of the anti-inflammatory adipokine adiponectin, macrophage infiltration and adipocyte death. In this sense, hypoxia constitutes an important factor in the development of obesity-associated comorbidities (55–57). Although hypoxia was proposed as a new potential risk factor for chronic inflammation in obesity in 2004 (58), its biological significance was not disentangled until 2008 when new methods for detection of oxygen levels in the adipose tissue were developed (59). Since then, multiple studies have directly evidenced a significant increase in hypoxic stress in the adipose tissue of different obese mouse models and humans (59–61).

The hypoxia-inducible factor 1 (HIF-1) acts as a master regulator of oxygen homeostasis regulating the expression levels of a wide variety of genes that stimulate erythropoiesis, angiogenesis, and glycolysis in a cell-specific pattern (62, 63). Increased HIF-1α gene expression in the subcutaneous adipose tissue of obese patients have been shown to be reduced after surgery-induced weight loss (64). Moreover, it is well established that levels of HIF-1α are elevated in the adipose tissues of obese mice, exerting important roles in the development of obesity and insulin resistance (65, 66). Reportedly, *Hif1a* deficient mice under a high-fat diet exhibited beneficial effects in adipose tissue inflammation and insulin resistance development (67). Interestingly, an opposite role for Hif-2α has been proposed. The deletion of *Hif2a* in adipocytes exerts protective effects by reducing the expression of inflammation- and fibrosis-related genes and also counteracting Hif-1α effects (67).

Indeed, hypoxia is also a hallmark of cancer (68, 69). Hypoxia plays a main role in cancer by promoting changes in the tumoral microenvironment, inducing angiogenesis, sustained proliferative signaling, unlimited replicative potential, genetic instability and altering the metabolism of tumoral cells (70). Hypoxia induces a transcription programme that promotes an aggressive tumor phenotype by the activation of HIF-1 (71). Accumulated evidence has highlighted a strong link between hypoxia-induced inflammation and intestinal diseases including ulcerative colitis, intestinal ischemia, and CRC (72). Similar to obesity, HIF-1α and HIF-2α have divergent roles in colon cancer development (73). HIF1A overexpression was significantly associated with poor prognosis and higher colorectal cancer-specific mortality, whereas HIF-2α was not related with clinical outcome and exhibited an inverse association with high tumor grade and obesity (74). Xenograft studies have revealed that loss of expression of HIF-1α inhibited tumor growth and, in contrast, deficiency of HIF-2α stimulated tumoral cell proliferation (75). The potential of targeting hypoxia and HIFs as a possible therapy for different types of cancer has widely analyzed because of their significant involvement in cancer development (76, 77).

Importantly, apart from HIF-1 other transcription factors are involved in the response to hypoxia, including nuclear factor κ-B (NF-κB), cyclic AMP response element binding protein (CREB), activating protein-1 (AP-1), early growth response-1 (EGR-1) and p53 (56). NF-κB is a central transcriptional mediator of the inflammatory response and its activation leads to a coordinated expression of genes involved in the control of innate immunity, inflammation as well as cellular stress responses and cell survival (78–80). Reportedly, NF-κB regulates gene expression levels of *Hif1a* under basal conditions and also during hypoxia in animal models, linking inflammation and the hypoxic response (81). Previous studies also demonstrated the dual regulation between NF-κB and HIF-1 in cellular cultures (82, 83) Hypoxia also potentiates the NF-κB signaling by modulating the expression of toll like receptors (TLRs), which are known for their functional roles in the inflammatory response in both, infectious and non-infectious diseases and in the production of IL-1β, a main pro-inflammatory cytokine (55, 84).

## Inflammasome Biology and Activation

The innate immune system include a wide range of pattern-recognition receptors (PRRs) for sensing cellular insults including the nucleotide-binding oligomerization domain (NOD)-like receptors (NLRs), the retinoic acid-inducible gene I-like receptors (RLRs) and the toll-like receptors (TLRs) (53, 85). Among them, NLRs recognize diverse set of inflammation-inducing stimuli that include pathogen-associated molecular patterns (PAMPs) as well as host-derived danger signals (danger-associated molecular patterns, DAMPs) (53). Inflammasomes are intracellular multiprotein complexes that recognize both PAMPs and DAMPs. The essential components of inflammasomes comprise a sensor protein (NLR and PYHIN families), the adaptor apoptosis-associated speck-like protein containing a CARD (ASC) and the effector protease caspase-1. Hence, inflammasomes act as high-molecular weight platforms for caspase-1 activation (86) and once activated, caspase-1 triggers the maduration and release of important proinflammatory mediators such as IL-1β and IL-18 (53, 87). IL-1β is involved in the regulation of broadly systemic and local responses to infection and injury and, therefore, a tight control of its production is required (88, 89). In addition, inflammasomes also trigger the secretion of multiple proteins to coordinate cell proliferation and tissue repair such as fibroblast growth factor (FGF)-2, high mobility group box 1 (HMGB1), galectin-1, galectin-3, and IL-1α through an unconventional pathway (90, 91). Moreover, inflammasomes are required to coordinate other important mechanisms of inflammation and tissue repair including (i) pyroptosis, a type of lytic programmed cell death mediated by activation of caspase-1 and (ii) necrosis, a passive cell death resulting from external factors (92–94). Inflammasomes-induced processes are of great importance not only in the antimicrobial response but also in governing metabolic pathways and mucosal immune responses. An altered inflammasome function has been shown to be implicated in the pathogenesis of human diseases (92, 94).

According to their main constituent, inflammasomes can be categorized in two main groups: (i) the NLR family that includes the members NLRP1, NLRP2, NLRP3, NLRP6, NLRC4 and, potentially, NLRP12 and (ii) the PYHIN family that comprises the AIM2 and IFI16 members (95, 96). Among them, the NLRP3 inflammasome is the best characterized. Unlike other inflammasomes, the endogenous expression of NLRP3 in inactive immune cells is not enough to allow its activation representing a key limiting factor for its regulation (97). These evidences show that the activation of the NLRP3 inflammasome depends on a two-signal step process with a minimum of two stimuli being needed (98); first a priming step is required followed by a second activation step to induce the NLRP3 inflammasome formation (99, 100). The priming step can be mediated through PRRs, toll-like receptors (TLR), cytokine receptors or other factors upstream the nuclear factor NF-κB pathway (99–101) and then, the NLRP3 inflammasome assembles in response to a wide array of signals including PAMPs, bacterial toxins and DAMPs (ATP, uric acid, amyloid β fibrils, cholesterol crystals ceramides, palmitate and hyaluronan) (102–104). Saturated fatty acids induce the activation of the NLRP3 inflammasome in macrophages by increasing mitochondrial reactive oxygen species as well as by activating the AMP-activated protein kinase and autophagy signaling cascades (105, 106). Hypoxia has been also described to be involved in the regulation of NLRP3 activation in adipocytes. A recent study has elegantly demonstrated that the increased gene expression levels of the NLRP3 inflammasome components induced by homocystein, were downregulated in the epididymal adipose tissue of an adipocyte-specific *Hif1a* knockout mice (107). Moreover, reduced circulating levels of IL-1β and active caspase-1 were found in both adipocytes and macrophages infiltrating adipose tissue (107). Extracellular ATP is another key DAMP that induces NLRP3 inflammasome formation. The mechanism for producing ATP is dependent on the levels of O_2_ and regulated by HIF-1, that directs cells to produce ATP via glycolysis during O_2_ deprivation (54, 63, 108). Therefore, the increase in ATP under hypoxia conditions might be also involved in the activation of the NLRP3 inflammasome. While TLRs recognize PAMPs in the extracellular component and endosomes, NLRs act in the cytoplasm (109). Recent evidences have connected the NLRP3 inflammasome and TLRs in immune and metabolic regulation (108). The activation of TLRs offers a priming signal for inflammasome activation independent from the transcription of NLRP3 and involving critical factors such as IRAK1 and TRAF6 (110–113). Moreover, LPS has been shown to induce the expression of HIF-1α by a TLR4-dependent pattern in macrophages and in turn, HIF-1α up-regulated TLR4 expression under hypoxic stress (108, 114, 115), suggesting that both NLRP3 and TLR4 are under hypoxia control. The role of hypoxia, mitochondrial reactive oxygen species as well as glucose and lipid metabolism in NLRP3 activation and dysregulation has been exhaustively reviewed supporting their high potential in the design of innovative therapies for inflammasome-associated diseases (108, 116).

Therefore, NLRP3 activation is tightly regulated at multiple levels highlighting the significance of this protein complex in the development and progression of chronic inflammatory diseases and its potential for designing innovative therapeutic strategies (92, 94). Notably, recent studies have linked the activation of the NLRP3 inflammasome to different metabolic diseases including obesity, metabolic syndrome, T2D and cardiovascular alterations and the role of NLRP3 in cancer, especially in inflammation-induced cancers, has been extensively explored (101). However, the molecular pathways underlying the organ- and cell-specific activation of the NLRP3 inflammasome in the context of obesity have just stated to be elucidated.

## NLRP3 in the Development of Obesity and Insulin Resistance

Immune and metabolic responses must be tightly regulated for normal cellular homeostasis and both are highly conserved across species and throughout evolution (117). The NLRP3 inflammasome was first suggested to regulate adiposity and insulin sensitivity during obesity by Zhou et al. (118) demonstrating that mice lacking *Nlrp3* showed improved glucose homeostasis under a high fat diet (119). A novel study corroborated this finding reporting that *Nlrp3-, Caspase-1-*, or *Il1b*-knockout mice under a normal diet exhibited a reduction in their weight gain and fat mass, as well as an improvement in insulin sensitivity. These data suggest that the Nlrp3 inflammasome is involved in the regulation of glucose homeostasis (120). Indeed, mice lacking *Nlrp3* were resistant to the development of high fat diet-induced obesity and were also protected from obesity-induced insulin resistance. In this knockout mice model, the impaired inflammasome activation was associated with a reduction in the expression of the proinflammatory factor MCP-1 in adipose tissue as well as with a decrease in the size of adipocytes, in the number of infiltrated macrophages in adipose tissue and in the content of triglycerides in the liver (121). The improved metabolic phenotype of *Nlrp3*^−/−^ mice has been also linked to an attenuated *Il1b* expression in adipose tissue and lower serum concentrations of IL-18 (122). These observations are partly paralleled in humans, in whom the expression of the different components of the NLRP3 inflammasome in adipose tissue is directly associated with body weight in obese individuals with T2D as well as with the severity of T2D (122). Furthermore, weight loss in obese patients with T2D was correlated with a significant downregulation in *NLRP3* and *IL1B* gene expression in subcutaneous adipose tissue. Another evidence for a pathogenic role of NLRP3 in T2D comes from a study reporting that glyburide, a sulfonylurea drug for the treatment of T2D, inhibits NLRP3-mediated IL-1β release in monocytes (123). In addition, the islet amyloid polypeptide (IAPP or amilin), a hallmark feature in the pancreas of most patients with T2D, triggeres IL-1β production via NLPR3 in lipopolysaccharide (LPS)-primed macrophages or dendritic cells (124).

Considering that the development of obesity is associated with hypoxia and adipocyte death, NLRP3 is preferentially expressed in ATMs from the crown-like structures. Lipotoxic ceramides and the saturated fatty acid palmitate can also trigger NLRP3 activation through a mechanism that involves defective autophagy and the accumulation of mitochondrial reactive oxygen species (ROS) (94, 106). Importantly, obesity is associated with increased circulating levels of palmitate, constituting one of the most abundant free fatty acids in the obese state (101). Obesity itself also promote the assembly of the NLRP3 inflammasome in ATMs, inducing macrophage-mediated T cell activation that, through the release of INF-γ, mediates insulin resistance (101, 122). In addition, the activation of caspase-1 in ATM induces t detrimental effects in insulin-sensitive tissues: (i) IL-1β impairs insulin signaling by direct phosphorylation of insulin receptor substrate 1 and by inducing the expression of TNF-α, a well-characterized pro-inflammatory and insulin resistance promoting cytokine; and (ii) IL-1β induces type 1 CD4 T helper cells in adipose tissue leading to lymphocyte accumulation and activation and promoting insulin resistance (94, 125). Despite unequivocal evidences causally link IL-1β to the development of obesity-associated comorbities, IL-18, also activated by caspase-1, has been shown to ameliorate the development of obesity and insulin resistance (126). Obese individuals have increased levels of circulating IL-18 that are markedly correlated with metabolic syndrome and insulin resistance (127–129). However, somewhat paradoxically, IL-18 has anti-obesity effects (129). *Il-18*- and *Il-18* receptor-deficient mice exhibit a notably increase in their body weight compared to wild-type littermates of the same age (126). Mice treated intracerebrally with recombinant IL-18 showed an inhibition in food intake as well as an improvement in glucose homesotasis (126). Recently, IL-18 production from the NLRP1 inflammasome has been associated to the prevention of obesity and metabolic syndrome (129). Reportedly, *Caspase-1* knockout mice also deficient in both IL-18 and IL-1β exhibited an improvement in glucose metabolism, suggesting that the insulin desensitizing effects of IL-1β override IL-18 action (126, 130, 131).

Besides its functions in ATMs, the NLRP3 inflammasome is also an important regulator of intrinsic adipocyte functions, controlling adipocyte differentiation. Caspase-1 is upregulated during adipocyte differentiation and, through the activation of IL-1β, directs adipocytes toward a more insulin-resistant phenotype. In addition, *Nlrp3*- and *Caspase-1*-deficient preadipocytes resulted in more metabolically efficient fat cells (120). Importantly, the loss of function of NLRP3 is associated with a decrease in the activation of caspase-1 but does not completely inhibit its function, suggesting that additional non-NLRP3 inflammasome sensors might be controlling its activation. This reveals the interesting possibility of specific inflammasome components present in macrophages, adipocytes, or pancreatic β cells inducing the development of obesity-associated low-grade chronic inflammation and hence, its associated comorbidities (130).

## Role of NLRP3 in Colorectal Cancer

The gastrointestinal mucosa homeostasis depends on complex interactions between diverse regulatory mechanisms: the microbiota, the intestinal epithelial cells and the host immune system with a breakdown in these pathways plausibly precipitating a chronic inflammatory pathology (94, 132). The ability of inflammasomes to recognize exogenous and endogenous pathogenic insults (91) has led to analyze their roles in intestinal inflammatory bowel diseases (IBD), including ulcerative colitis, Crohn's disease, and CRC development. However, conflicting results regarding the role of the NLRP3 inflammasome have been reported by several groups. Whereas, some studies have reported reduced disease severity in *Nlrp3* or *Caspase-1* knockout mice due to a lower IL-1β production, other groups have found that *Nlrp3-, Asc-* and *Caspase-1*-deficient mice showed an increased disease severity (133–136). Indeed, the cytokines IL-1β and IL-18, the main downstream signaling molecules from NLRP3 pathway, exert pleiotropic effects in inflammation and tumorigenesis with both, pro- and anti-tumorigenic functions having been extensively studied (137, 138). This seemingly contradictory function has been proposed to be context-dependent and tissue-specific. In an elegant study, ASC, a main component of the inflammasomes, was shown to promote tumor development favoring inflammation in infiltrating immune cells, and, oppositely, to limit the proliferation of cancer cells via the activation of p53 in epithelial cells (139). Thus, a model in which the hypo- or hyper-functionality of the inflammasome led to an imbalance of the intestinal homeostasis has been proposed. In this sense, the specific function of the inflammasome in the intestinal tissue depends on the affected cell type: the activation of the inflammasome is required for controlling epithelium permeability and regeneration but its excessive activation within the lamina propia promotes severe intestinal inflammation (140). Reportedly, the IL-22–IL-22 binding protein (IL-22BP) axis is critically involved in both, intestinal cellular renovation and colon tumor development. Colon tissue damage triggers the activation of the NLRP3 inflammasome that downregulates the expression levels of IL-2BP through the activation of IL-18 via caspase-1. It leads to an increase in the ratio of IL-22/IL-22BP allowing IL-22 exert its protective roles during the peak of damage. However, an uncontrolled and prolonged expression of IL-22 may promote CRC development (141). This observation suggests a critical role for IL-18 in colon carcinogenesis. In this regard, differential actions of IL-18 have been also described during the acute and chronic stages of colitis (142).

Relevant studies focused on the activity of the inflammasome in cancer development have demonstrated that NLRP3 is highly expressed in a cell line of mesenchymal-like colon cancer cells with its expression being upregulated by TNF-α and transforming growth factor-β1 (TGF-β1) during the epithelial-mesenchymal transition (143). In addition, the knockdown of *NLRP3* in colon carcinoma cells has been associated with a decreased capacity of migration and invasion (143). Furthermore, a high cholesterol diet has been shown to promote colon carcinogenesis in azoxymethane (AOM)-treated mice via the activation of the NLRP3 inflammasome (144). Consistently, cholesterol inhibited the activity of AMP-activated protein kinase (AMPK)-α in macrophages, increasing the production of mitochondrial ROS, which in turn activated the NLRP3 inflammasome (144). These data underline the influence of diet in controlling molecular pathways associated with chronic inflammatory disorders and CRC. The inhibition of the NLRP3 inflammasome in macrophages from an AOM-dextran sulfate sodium (DSS) mouse model of colon carcinoma significantly protected against colitis-associated cancer (145). Despite mounting evidence about its pro-carcinogenic activities, *NLRP3* deficiency has been linked not only to an increased number of colon polyps but also to a greater susceptibility to colon cancer development in an AOM-DSS mouse model, suggesting a protective role for NLRP3 in colon carcinogenesis (133). Recently, genome-wide association studies have linked single nucleotide polymorphisms in the gene encoding NLRP3 with increased susceptibility to Crohn's disease (146) as well as with poor survival rate for colorectal cancer (92, 147).

## IL-1β and IL-18 in Obesity and Colon Cancer

IL-1β is an “alarmin cytokine” with pleiotropic functions mainly produced and released by inflammatory or stress signals (148). A dysregulated expression of IL-1β has been linked to tumor development and growth in different types of cancer including CRC (149–153). In this sense, an increased IL-1β expression associated with a high-grade inflammation in colonic mucosa has been described in patients with IBD as well as in several animal models of the disease (154–157). IL-1β is also able to act on intestinal epithelial cells promoting epithelial-mesenchymal transition and hence, contributing to colon tumor cell invasiveness (158, 159). Moreover, IL-1β stimulates the production of reactive oxidative species that induce DNA damage and cancer development. An increased expression of the microRNA-301a promoted by IL-1β has been recently described in patients with CC. The microRNA-301a induced colon inflammation and cancer development by the production of strong pro-inflammatory cytokines that compromised colonic mucosa of patients with IBD (160). IL-1β also stimulates the expression of syndecan-2, not only a well-known colon cancer marker but also an inflammatory hypoxia marker, strengthening the relationship between chronic inflammation and the development of CC (161). Consistently, mutations in the IL-1β gene resulted in increased protein levels and therefore, in a higher risk of colon cancer development (162) and mutations in the nucleotide binding oligomerization domain containing (NOD)-2 gene have been shown to increase NF-κB activity and IL-1β processing in patients with severe forms of Crohn's disease, indicating an association between IL-1β and the development of IBD (163). Further studies in *Il1b*-deficent mice have shown an impairment of the potent IL-1β-mediated inflammation limiting tumor development (152, 155).

Tumor-associated macrophages (TAM) are key effectors in cancer-related inflammation and reportedly, activated macrophages secrete IL-1β resulting in the activation of NF-κB signaling favoring tumorigenic progression (164, 165). In this line, the AW264.7 macrophage cell line is unable to secrete the mature form of IL-1β because of the absence of a specific component of the inflammasome, the caspase-activating recruiting domain (ASC), suggesting the existence of different release pathways (166). The secretion of IL-1β by macrophages is also required to activate Wnt signaling and β-catenin/TCF4 transcriptional activity in tumor cells promoting tumor growth (167–169). Obesity-associated adipose tissue inflammation is characterized by an infiltration of M1 activated macrophages. Based on growing evidence, excess adipose tissue has been linked to cancer promotion by its capacity to recruit pro-inflammatory macrophages that release pro-inflammatory cytokines with pro-carcinogenic effects including IL-1β as well as by the great variety of inflammatory adipokines synthesized by the adipocytes (40, 41). In obesity-associated breast cancer, adipocytes are able to recruit macrophages by a novel signaling pathway involving IL-1β and the chemotactic factor CCL2 (also known as MCP-1) that in turn activate CXCL12 promoting angiogenesis and tumor development (170). The relationship of CCL2 and IL-1β has been previously reported since *Ccl2* knockout mice exhibited a reduced angiogenesis induced by IL-1β (171).

IL-18 also known as interferon-γ inducing factor (IGIF) is a central cytokine in the regulation of both innate and acquired immune responses (172, 173). IL-18 also exhibits lymphocyte chemoattractant properties and induces the expression of pro-inflammatory mediators as well as angiogenesis- and adhesion-related factors (174). IL-18 is widely overexpressed in patients with IBD, especially in patients with Crohn's disease (155, 175). Furthermore, gene and circulating expression levels of IL-18 are increased in gastrointestinal-associated cancers (176). However, IL-18 has a dual activity in the pathogenesis of CC (155). Whereas, during early stages of CC IL-18 exerts a protective role including epithelial repair processes (177) during later stages, it promotes tumor growth and cellular invasion (178). Significantly, the importance of cell specificity for IL-18 signaling in colitis has been previously described (179).

Obesity and obesity-associated insulin resistance have been also associated with increased circulating IL-18 levels (180, 181). In accordance, a hypocaloric diet induced a reduction in plasma IL-18 levels (182). Although human adipocytes express IL-18, they are unlikely to contribute significant amounts of IL-18 into circulation and their increased levels associated with obesity (183). However, an independent study has demonstrated that human adipose tissue contributes to systemic IL-18 concentrations (184). Increased levels of IL-18 and TNF-α have been described in the colon of diet-induced obese mice in parallel with alterations in the Wnt signaling pathway suggesting a mechanism for obesity-associated CC development (185).

## NLRP3 Inflammasome in Obesity and Colon Cancer

**Figure 1:**
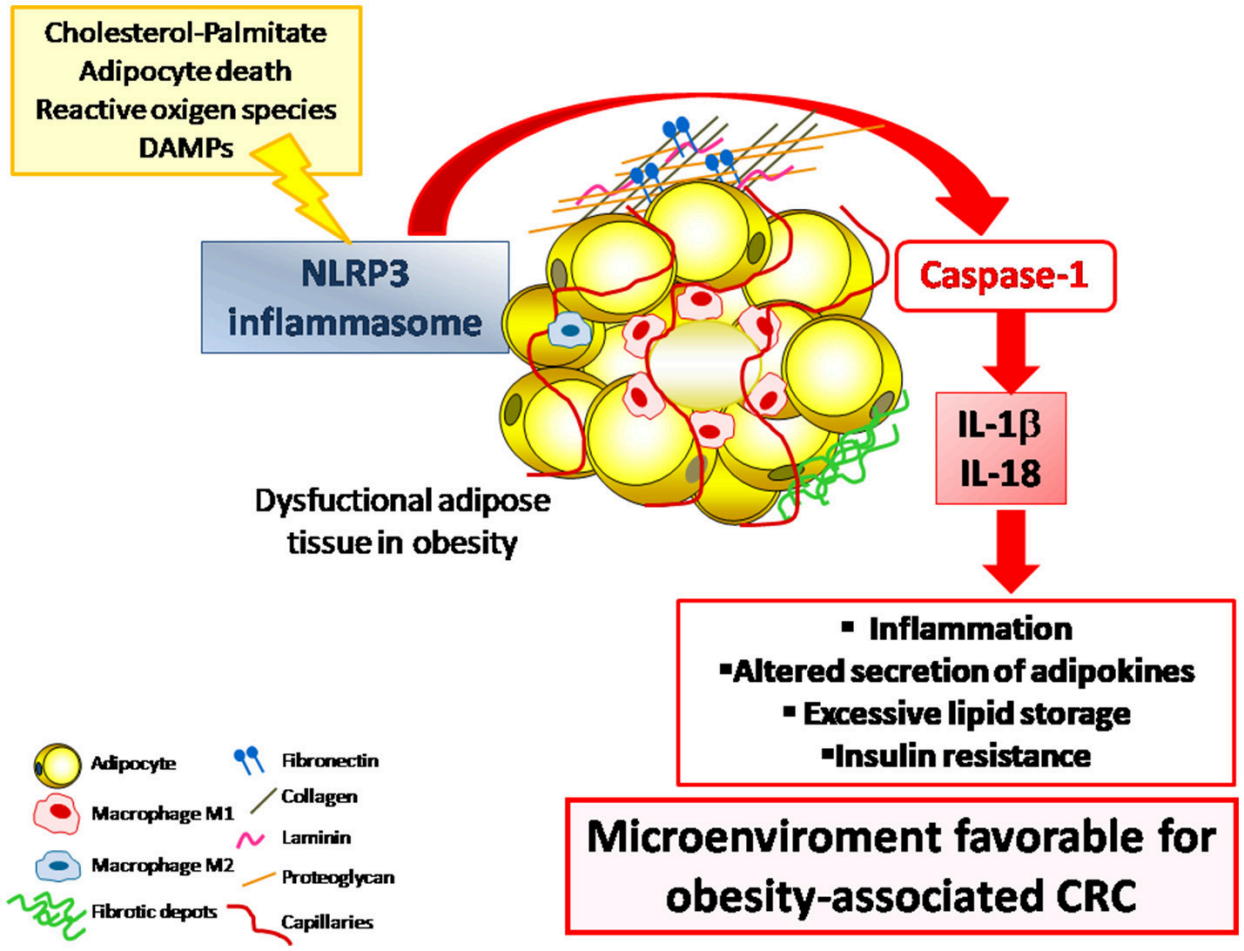
The NLRP3 inflammasome is a multiprotein intracellular complex that, in response to DAMPs and PAMPs, regulates the activation of caspase-1 and induces inflammation through the potent pro-inflammatory cytokines IL-1β and IL-18. The dysregulated expression of NLRP3 inflammasome in the adipose tissue in the obese state contributes to the development of a low-grade chronic inflammatory state that promotes a microenviroment that favors the development inflammation-associated diseases including CRC. CRC, colorectal cancer; DAMPs, damage-associated molecular patterns; IL, interleukin; NLRP3, NLR family pyrin domain containing 3; PAMPs, pathogen-associated molecular patterns.

Chronic inflammation and excessive adipose tissue accumulation are known risk factors for numerous chronic conditions including CRC (6, 69, 186). Whether obesity has an impact on CRC development by enhancing inflammatory signaling pathways or through a direct mechanism remain largely unclear and the role of NLRP3 in colon cancer is still controversial. A causal mechanism by which obesity promotes the progression of breast cancer via the NLRC4 inflammasome activation has been recently described (187) but no results showing the impact of NLRP3 in obesity-associated CRC have been reported. A possible mechanism in this association may involve the IL-1β pathway, strongly upregulated in obese patients with CRC (188). In this sense, IL-1β has been shown to promote CRC progression by inducing the release of inflammatory and extracellular matrix remodeling factors by acting directly on adipocytes or indirectly on macrophages (189) (Figure 1).

Obesity has been also broadly associated to poor prognosis of CRC by influencing tumor microenvironment through the release of pro-inflammatory adipokines by adipocytes and inflammatory factors by tumor-associated adipocytes (TAAs). TAAs are able to modulate and recruit TAM which, in turn, can promote tumor angiogenesis and metastatic spread (190). TAMs display an increased activation of the NLRP3 inflammasome, resulting in a significant increase in IL-1β expression that aggravates colon inflammation and favors CRC development. Importantly, improved survival as well as reduced lymph node invasion and distant metastasis have been reported for patients with invasive breast cancer with low levels of infiltrating NLRP3^+^ macrophages (191). These data sustain the concept that the enhanced expression of *NLRP3* in TAM correlates with disease aggressiveness and metastasis (191, 192). Further studies are required to fully explain the molecular pathways underlying the impact of NLRP3-IL-1β in obesity-associated CRC.

## Therapeutic Interventions

A huge body of knowledge supports the link between cancer and obesity, or more specifically with excess adiposity. Thus, it seems reasonable to translate these findings to the clinical practice and focus our attention more on adipose tissue by performing body composition analyses or at least estimating body fat by available validated equations (193). In this line, it is also important to gain more insight into the impact of adipobiology given its direct relationship with energy homeostasis (21, 194), inflammation (195), T2D-insulin resistance (196, 197) and lipid metabolism (198–200). Moreover, the influence of already well-known adipokines or as yet unidentified additional factors with cancer development should be considered (201–205).

The well-established inflammatory potential of NLRP3 together with its central role for immune sensing as well as its key function in different diseases makes NLRP3 an attractive therapy target. The agonists of NLRP3 may be useful in immunotherapy of specific solid tumors by enhancing the immune function to reverse the immunosuppressive microenvironment (206). For instance, the activation of IL-18 signaling by the inflammasome controls colon tissue regeneration following CRC by inducing a process of intestinal re-epithelialization (207). The inflammasome signaling is also involved in the regulation of immunosurveillance, which by its combination with chemotherapy treatments induce immunogenic cell death (208) and by its interactions with TLR pathways promote an efficient antitumoral immunity (209). Since the activation of NLRP3 drives pyroptotic and immunogenic cell death, its direct activation within the tumor has been described as an efficient mechanism to mediate a potent and persistent antitumor immunity (210). However, in specific conditions such as obesity, the chronic activation of NLRP3 can promote tumor cell growth, migration and invasion (97). Thus, while long-term chronic inflammation may constitute a mechanism to support the growth of several tumors, the acute and robust activation of NLRP3 may have the capacity to activate the immune system for tumor destruction (97).

On the other hand, pharmacological inhibitors of the NLRP3 pathway could offer a better treatment option in a wide array of chronic- and auto-inflammatory diseases for which no adequate therapies exist (97). The development of NLRP3 antagonists constitutes an important research topic since only a specific subset of NLRP3-associated pathologies can be efficiently treated with agents that block the signaling pathway of IL-1β, including (i) anakinra, a recombinant and slightly modified version of the IL-1 receptor antagonist, (ii) canakinumab, a monoclonal antibody to neutralize IL-1β, and (iii) rilonacept, a long-acting soluble decoy IL-1β receptor engineered as a dimeric fusion protein (211). Different NLRP3 inflammasome inhibitors, including sulforaphane, β-hydroxybutyrate, glyburide, flufenamic acid, mefenamic acid, parthenolide, BAY 11-7082, INF39, and MCC950 have been developed, but there is no evidence demonstrating that these compounds directly and specifically target NLRP3 itself (212). Among these compounds, BAY 11-7082 has been shown to attenuate the increase of the NLRP3 inflammasome expression after a high fat diet leading to the repression of caspase-1 activation as well as to the reduction of IL-1β and IL-18 release, normalizing the diet-induced impairment of metabolic parameters (213). The fasting- or exercise-induced ketone metabolite β-hydroxybutyrate exhibited the potential to inhibit the NLRP3 inflammasome in human macrophages dampening innate immune responses and sparing ATP for the function of ketone-dependent organs. These results suggest that dietary approaches to increase the β-hydroxybutyrate levels may be a promise in decreasing the severity of NLRP3-associated chronic inflammatory diseases (214). However, the direct target of NLRP3 has potentially increased efficacy and decreased side effects over these current treatments. Recently, CY-09 has been reported to directly target NLRP3, inhibiting its activation *in vivo* and reversing metabolic disorders through inhibition of NLRP3-dependent IL-1β production in a mouse model of T2D (212, 214). Further research characterizing the molecular mechanisms in which these compounds are involved will help us to establish the specific functions of NLRP3.

In light of the fact that the prevalence of obesity and CRC is increasing in an alarming rate, a more detailed studies identifying the roles and mechanisms of action of NLRP3 as well as the clinical development of molecules to selectively antagonize the NLRP3 inflammasome may lead to a better understanding of the pathogenesis of obesity-associated comorbidities as well as to the develop of innovative precision medicine approaches for the management of obesity and its complications (215, 216).

## Author Contributions

PA, GF, and VC: wrote and edited the manuscript; GZ, PM, JH-L, JB and XU: contributed to writing and critically revised the paper; VC and GF: designed the figure. All authors read, corrected, and approved the final manuscript.

### Conflict of Interest Statement

The authors declare that the research was conducted in the absence of any commercial or financial relationships that could be construed as a potential conflict of interest.

## References

[1] Kuipers EJ Grady WM Lieberman D Seufferlein T Sung JJ Boelens PG. Colorectal cancer. Nat Rev Dis Primers (2015) 1:15065. 10.1038/nrdp.2015.65 27189416 PMC4874655

[2] Siegel RL Miller KD Jemal A Cancer statistics, 2018. CA Cancer J Clin. (2018) 68:7–30. 10.3322/caac.21442 29313949

[3] O'Keefe SJ. Diet, microorganisms and their metabolites, and colon cancer. Nat Rev Gastroenterol Hepatol. (2016) 13:691–706. 10.1038/nrgastro.2016.165 27848961 PMC6312102

[4] Parekh N Chandran U Bandera EV. Obesity in cancer survival. Annu Rev Nutr. (2012) 32:311–42. 10.1146/annurev-nutr-071811-150713 22540252 PMC3807693

[5] Park J Euhus DM Scherer PE. Paracrine and endocrine effects of adipose tissue on cancer development and progression. Endocr Rev. (2011) 32:550–70. 10.1210/er.2010-0030 21642230 PMC3369575

[6] Park J Morley TS Kim M Clegg DJ Scherer PE. Obesity and cancer-mechanisms underlying tumour progression and recurrence. Nat Rev Endocrinol. (2014) 10:455–65. 10.1038/nrendo.2014.94 24935119 PMC4374431

[7] Schwartz B Yehuda-Shnaidman E. Putative role of adipose tissue in growth and metabolism of colon cancer cells. Front Oncol. (2014) 4:164. 10.3389/fonc.2014.00164 25019059 PMC4071563

[8] Larsson SC Wolk A. Obesity and colon and rectal cancer risk: a meta-analysis of prospective studies. Am J Clin Nutr. (2007) 86:556–65. 10.1093/ajcn/86.3.556 17823417

[9] Moghaddam AA Woodward M Huxley R. Obesity and risk of colorectal cancer: a meta-analysis of 31 studies with 70,000 events. Cancer Epidemiol Biomarkers Prev. (2007) 16:2533–47. 10.1158/1055-9965.EPI-07-0708 18086756

[10] Renehan AG Tyson M Egger M Heller RF Zwahlen M. Body-mass index and incidence of cancer: a systematic review and meta-analysis of prospective observational studies. Lancet. (2008) 371:569–78. 10.1016/S0140-6736(08)60269-X 18280327

[11] Giovannucci E Michaud D. The role of obesity and related metabolic disturbances in cancers of the colon, prostate, and pancreas. Gastroenterology (2007) 132:2208–25. 10.1053/j.gastro.2007.03.050 17498513

[12] Sung MK Yeon JY Park SY Park JH Choi MS. Obesity-induced metabolic stresses in breast and colon cancer. Ann N Y Acad Sci. (2011) 1229:61–8. 10.1111/j.1749-6632.2011.06094.x 21793840

[13] World Cancer Research Fund/American Institute for Cancer Research Continuous Update Project Report. Food, Nutrition, Physical Activity and the Prevention of Colorectal Cancer. (2011). Available online at: www.dietandcancerreport.org/.

[14] Eddi R Karki A Shah A DeBari VA DePasquale JR. Association of type 2 diabetes and colon adenomas. J Gastrointest Cancer (2012) 43:87–92. 10.1007/s12029-011-9316-7 21894459

[15] Gonzalez N Prieto I Del Puerto-Nevado L Portal-Nunez S Ardura JA Corton M. 2017 update on the relationship between diabetes and colorectal cancer: epidemiology, potential molecular mechanisms and therapeutic implications. Oncotarget (2017) 8:18456–85. 10.18632/oncotarget.14472 28060743 PMC5392343

[16] Guraya SY. Association of type 2 diabetes mellitus and the risk of colorectal cancer: a meta-analysis and systematic review. World J Gastroenterol (2015) 21:6026–31. 10.3748/wjg.v21.i19.6026 26019469 PMC4438039

[17] NCD Risk Factor Collaboration (NCD-RisC) Worldwide trends in body-mass index, underweight, overweight, and obesity from 1975 to 2016: a pooled analysis of 2416 population-based measurement studies in 128.9 million children, adolescents, and adults. Lancet (2017) 390:2627–42. 10.1016/S0140-6736(17)32129-3 29029897 PMC5735219

[18] Collaborators TGO Health effects of overweight and obesity in 195 countries over 25 years. N Engl J Med. (2017) 377:13–27. 10.1056/NEJMoa1614362 28604169 PMC5477817

[19] Villarejo C Fernandez-Aranda F Jimenez-Murcia S Penas-Lledo E Granero R Penelo E. Lifetime obesity in patients with eating disorders: increasing prevalence, clinical and personality correlates. Eur Eat Disord Rev. (2012) 20:250–4. 10.1002/erv.2166 22383308 PMC3510304

[20] Jensen MD. Role of body fat distribution and the metabolic complications of obesity. J Clin Endocrinol Metab. (2008) 93:S57–63. 10.1210/jc.2008-1585 18987271 PMC2585758

[21] Rodríguez A Ezquerro S Méndez-Gimenez L Becerril S Frühbeck G. Revisiting the adipocyte: a model for integration of cytokine signaling in the regulation of energy metabolism. Am J Physiol Endocrinol Metab. (2015) 309:E691–714. 10.1152/ajpendo.00297.2015 26330344

[22] Moore LL Chadid S Singer MR Kreger BE Denis GV. Metabolic health reduces risk of obesity-related cancer in framingham study adults. Cancer Epidemiol Biomarkers Prev. (2014) 23:2057–65. 10.1158/1055-9965.EPI-14-0240 25012997 PMC4184957

[23] Calle EE Kaaks R. Overweight, obesity and cancer: epidemiological evidence and proposed mechanisms. Nat Rev Cancer (2004) 4:579–91. 10.1038/nrc1408 15286738

[24] Catalán V Gómez-Ambrosi J Rodríguez A Frühbeck G. Adipose tissue immunity and cancer. Front Physiol. (2013) 4:275. 10.3389/fphys.2013.00275 24106481 PMC3788329

[25] Pérez-Hernandez AI Catalán V Gómez-Ambrosi J Rodríguez A Frühbeck G. Mechanisms linking excess adiposity and carcinogenesis promotion. Front Endocrinol. (2014) 5:65. 10.3389/fendo.2014.00065 24829560 PMC4013474

[26] van Kruijsdijk RC van der Wall E Visseren FL. Obesity and cancer: the role of dysfunctional adipose tissue. Cancer Epidemiol Biomarkers Prev. (2009) 18:2569–78. 10.1158/1055-9965.EPI-09-0372 19755644

[27] Howe LR Subbaramaiah K Hudis CA Dannenberg AJ. Molecular pathways: adipose inflammation as a mediator of obesity-associated cancer. Clin Cancer Res. (2013) 19:6074–83. 10.1158/1078-0432.CCR-12-2603 23958744 PMC3891839

[28] Osborn O Olefsky JM. The cellular and signaling networks linking the immune system and metabolism in disease. Nat Med. (2012) 18:363–74. 10.1038/nm.2627 22395709

[29] Unamuno X Gómez-Ambrosi J Rodríguez A Becerril S Frühbeck G Catalán V. Adipokine dysregulation and adipose tissue inflammation in human obesity. Eur J Clin Invest. (2018) 48:e12997. 10.1111/eci.12997 29995306

[30] Iyengar NM Gucalp A Dannenberg AJ Hudis CA. Obesity and cancer mechanisms: tumor microenvironment and inflammation. J Clin Oncol. (2016) 34:4270–6. 10.1200/JCO.2016.67.4283 27903155 PMC5562428

[31] Zitvogel L Pietrocola F Kroemer G. Nutrition, inflammation and cancer. Nat Immunol. (2017) 18:843–50. 10.1038/ni.3754 28722707

[32] Balkwill F Mantovani A. Inflammation and cancer: back to Virchow? Lancet (2001) 357:539–45. 10.1016/S0140-6736(00)04046-0 11229684

[33] Flores BM O'Connor A Moss AC. Impact of mucosal inflammation on risk of colorectal neoplasia in patients with ulcerative colitis: a systematic review and meta-analysis. Gastrointest Endosc. (2017) 86:1006–11 e8. 10.1016/j.gie.2017.07.028 28750838

[34] Grivennikov SI. Inflammation and colorectal cancer: colitis-associated neoplasia. Semin Immunopathol. (2013) 35:229–44. 10.1007/s00281-012-0352-6 23161445 PMC3568220

[35] Rutter M Saunders B Wilkinson K Rumbles S Schofield G Kamm M. Severity of inflammation is a risk factor for colorectal neoplasia in ulcerative colitis. Gastroenterology (2004) 126:451–9. 10.1053/j.gastro.2003.11.010 14762782

[36] Yehuda-Shnaidman E Schwartz B. Mechanisms linking obesity, inflammation and altered metabolism to colon carcinogenesis. Obes Rev. (2012) 13:1083–95. 10.1111/j.1467-789X.2012.01024.x 22937964

[37] Balkwill F Charles KA Mantovani A. Smoldering and polarized inflammation in the initiation and promotion of malignant disease. Cancer Cell (2005) 7:211–7. 10.1016/j.ccr.2005.02.013 15766659

[38] Iyengar NM Hudis CA Dannenberg AJ. Obesity and cancer: local and systemic mechanisms. Annu Rev Med. (2015) 66:297–309. 10.1146/annurev-med-050913-022228 25587653

[39] Lumeng CN Bodzin JL Saltiel AR. Obesity induces a phenotypic switch in adipose tissue macrophage polarization. J Clin Invest. (2007) 117:175–84. 10.1172/JCI29881 17200717 PMC1716210

[40] McNelis JC Olefsky JM. Macrophages, immunity, and metabolic disease. Immunity (2014) 41:36–48. 10.1016/j.immuni.2014.05.010 25035952

[41] Font-Burgada J Sun B Karin M. Obesity and cancer: the oil that feeds the flame. Cell Metab. (2016) 23:48–62. 10.1016/j.cmet.2015.12.015 26771116

[42] Agrawal D Chen T Irby R Quackenbush J Chambers AF Szabo M. Osteopontin identified as lead marker of colon cancer progression, using pooled sample expression profiling. J Natl Cancer Inst. (2002) 94:513–21. 10.1093/jnci/94.7.513 11929952

[43] Catalán V Gómez-Ambrosi J Ramírez B Rotellar F Pastor C Silva C. Proinflammatory cytokines in obesity: impact of type 2 diabetes mellitus and gastric bypass. Obes Surg. (2007) 17:1464–74. 10.1007/s11695-008-9424-z 18219773

[44] Catalán V Gómez-Ambrosi J Rodríguez A Ramírez B Izaguirre M Hernández-Lizoain JL. Increased obesity-associated circulating levels of the extracellular matrix proteins osteopontin, chitinase-3 like-1 and tenascin C are associated with colon cancer. PLoS ONE (2016) 11:e0162189. 10.1371/journal.pone.0162189 27612200 PMC5017763

[45] Catalán V Gómez-Ambrosi J Rodríguez A Ramírez B Ortega VA Hernández-Lizoain JL. IL-32α-induced inflammation constitutes a link between obesity and colon cancer. Oncoimmunology (2017) 6:e1328338. 10.1080/2162402X.2017.1328338 28811968 PMC5543901

[46] Shao R Hamel K Petersen L Cao QJ Arenas RB Bigelow C. YKL-40, a secreted glycoprotein, promotes tumor angiogenesis. Oncogene (2009) 28:4456–68. 10.1038/onc.2009.292 19767768 PMC2795793

[47] Weisberg SP McCann D Desai M Rosenbaum M Leibel RL Ferrante AW Jr. Obesity is associated with macrophage accumulation in adipose tissue. J Clin Invest. (2003) 112:1796–808. 10.1172/JCI200319246 14679176 PMC296995

[48] de Visser KE Eichten A Coussens LM. Paradoxical roles of the immune system during cancer development. Nat Rev Cancer (2006) 6:24–37. 10.1038/nrc1782 16397525

[49] Greten FR Eckmann L Greten TF Park JM Li ZW Egan LJ. IKKβ links inflammation and tumorigenesis in a mouse model of colitis-associated cancer. Cell (2004) 118:285–96. 10.1016/j.cell.2004.07.013 15294155

[50] Di Caro G Marchesi F Laghi L Grizzi F. Immune cells: plastic players along colorectal cancer progression. J Cell Mol Med. (2013) 17:1088–95. 10.1111/jcmm.12117 24151976 PMC4118167

[51] Palucka AK Coussens LM. The basis of oncoimmunology. Cell (2016) 164:1233–47. 10.1016/j.cell.2016.01.049 26967289 PMC4788788

[52] Petrilli V. The multifaceted roles of inflammasome proteins in cancer. Curr Opin Oncol. (2017) 29:35–40. 10.1097/CCO.0000000000000346 27875342

[53] Schroder K Tschopp J. The inflammasomes. Cell (2010) 140:821–32. 10.1016/j.cell.2010.01.040 20303873

[54] Semenza GL. Life with oxygen. Science (2007) 318:62–4. 10.1126/science.1147949 17916722

[55] Eltzschig HK Carmeliet P. Hypoxia and inflammation. N Engl J Med. (2011) 364:656–65. 10.1056/NEJMra0910283 21323543 PMC3930928

[56] Trayhurn P. Hypoxia and adipose tissue function and dysfunction in obesity. Physiol Rev. (2013) 93:1–21. 10.1152/physrev.00017.2012 23303904

[57] Ye J. Emerging role of adipose tissue hypoxia in obesity and insulin resistance. Int Jf Obes. (2009) 33:54–66. 10.1038/ijo.2008.229 19050672 PMC2650750

[58] Trayhurn P Wood IS. Adipokines: inflammation and the pleiotropic role of white adipose tissue. Br J Nutr. (2004) 92:347–55. 10.1079/BJN20041213 15469638

[59] Trayhurn P Wang B Wood IS. Hypoxia in adipose tissue: a basis for the dysregulation of tissue function in obesity? Br J Nutr. (2008) 100:227–35. 10.1017/S0007114508971282 18397542

[60] Hosogai N Fukuhara A Oshima K Miyata Y Tanaka S Segawa K. Adipose tissue hypoxia in obesity and its impact on adipocytokine dysregulation. Diabetes (2007) 56:901–11. 10.2337/db06-0911 17395738

[61] Ye J Gao Z Yin J He Q. Hypoxia is a potential risk factor for chronic inflammation and adiponectin reduction in adipose tissue of ob/ob and dietary obese mice. Am J Physiol Endocrinol Metab. (2007) 293:E1118–28. 10.1152/ajpendo.00435.2007 17666485

[62] Semenza GL. HIF-1 and mechanisms of hypoxia sensing. Curr Opin Cell Biol. (2001) 13:167–71. 10.1016/S0955-0674(00)00194-0 11248550

[63] Semenza GL. HIF-1, O(2), and the 3 PHDs: how animal cells signal hypoxia to the nucleus. Cell (2001) 107:1–3. 10.1016/S0092-8674(01)00518-9 11595178

[64] Cancello R Henegar C Viguerie N Taleb S Poitou C Rouault C. Reduction of macrophage infiltration and chemoattractant gene expression changes in white adipose tissue of morbidly obese subjects after surgery-induced weight loss. Diabetes (2005) 54:2277–86. 10.2337/diabetes.54.8.2277 16046292

[65] Jiang C Qu A Matsubara T Chanturiya T Jou W Gavrilova O. Disruption of hypoxia-inducible factor 1 in adipocytes improves insulin sensitivity and decreases adiposity in high-fat diet-fed mice. Diabetes (2011) 60:2484–95. 10.2337/db11-0174 21873554 PMC3178277

[66] Lee KY Gesta S Boucher J Wang XL Kahn CR. The differential role of Hif1β/Arnt and the hypoxic response in adipose function, fibrosis, and inflammation. Cell Metab. (2011) 14:491–503. 10.1016/j.cmet.2011.08.006 21982709 PMC3206000

[67] Lee YS Kim JW Osborne O Oh DY Sasik R Schenk S. Increased adipocyte O2 consumption triggers HIF-1α, causing inflammation and insulin resistance in obesity. Cell (2014) 157:1339–52. 10.1016/j.cell.2014.05.012 24906151 PMC4114226

[68] Hanahan D Weinberg RA. The hallmarks of cancer. Cell (2000) 100:57–70. 10.1016/S0092-8674(00)81683-9 10647931

[69] Hanahan D Weinberg RA. Hallmarks of cancer: the next generation. Cell (2011) 144:646–74. 10.1016/j.cell.2011.02.013 21376230

[70] Harris AL. Hypoxia–a key regulatory factor in tumour growth. Nat Rev Cancer (2002) 2:38–47. 10.1038/nrc704 11902584

[71] Bertout JA Patel SA Simon MC. The impact of O2 availability on human cancer. Nat Rev Cancer (2008) 8:967–75. 10.1038/nrc2540 18987634 PMC3140692

[72] Bowser JL Phan LH Eltzschig HK. The hypoxia-adenosine link during intestinal inflammation. J Immunol. (2018) 200:897–907. 10.4049/jimmunol.1701414 29358413 PMC5784778

[73] Vadde R Vemula S Jinka R Merchant N Bramhachari PV Nagaraju GP. Role of hypoxia-inducible factors (HIF) in the maintenance of stemness and malignancy of colorectal cancer. Crit Rev Oncol Hematol. (2017) 113:22–7. 10.1016/j.critrevonc.2017.02.025 28427511

[74] Baba Y Nosho K Shima K Irahara N Chan AT Meyerhardt JA. HIF1A overexpression is associated with poor prognosis in a cohort of 731 colorectal cancers. Am J Pathol. (2010) 176:2292–301. 10.2353/ajpath.2010.090972 20363910 PMC2861094

[75] Imamura T Kikuchi H Herraiz MT Park DY Mizukami Y Mino-Kenduson M. HIF-1α and HIF-2α have divergent roles in colon cancer. Int J Cancer. (2009) 124:763–71. 10.1002/ijc.24032 19030186 PMC2682346

[76] Moyer MW. Targeting hypoxia brings breath of fresh air to cancer therapy. Nat Med. (2012) 18:636–7. 10.1038/nm0512-636b 22561804

[77] Wilson WR Hay MP. Targeting hypoxia in cancer therapy. Nat Rev Cancer (2011) 11:393–410. 10.1038/nrc3064 21606941

[78] Chen F Castranova V Shi X Demers LM. New insights into the role of nuclear factor-κB, a ubiquitous transcription factor in the initiation of diseases. Clin Chem. (1999) 45:7–17. 9895331

[79] Cummins EP Taylor CT. Hypoxia-responsive transcription factors. Pflugers Arch. (2005) 450:363–71. 10.1007/s00424-005-1413-7 16007431

[80] Barnes PJ Karin M. Nuclear factor-κB: a pivotal transcription factor in chronic inflammatory diseases. N Engl J Med. (1997) 336:1066–71. 10.1056/NEJM199704103361506 9091804

[81] Rius J Guma M Schachtrup C Akassoglou K Zinkernagel AS Nizet V. NF-κB links innate immunity to the hypoxic response through transcriptional regulation of HIF-1α. Nature (2008) 453:807–11. 10.1038/nature06905 18432192 PMC2669289

[82] Belaiba RS Bonello S Zahringer C Schmidt S Hess J Kietzmann T. Hypoxia up-regulates hypoxia-inducible factor-1α transcription by involving phosphatidylinositol 3-kinase and nuclear factor κB in pulmonary artery smooth muscle cells. Mol Biol Cell. (2007) 18:4691–7. 10.1091/mbc.e07-04-0391 17898080 PMC2096613

[83] Walmsley SR Print C Farahi N Peyssonnaux C Johnson RS Cramer T. Hypoxia-induced neutrophil survival is mediated by HIF-1α -dependent NF-κB activity. J Exp Med. (2005) 201:105–15. 10.1084/jem.20040624 15630139 PMC2212759

[84] Kuhlicke J Frick JS Morote-Garcia JC Rosenberger P Eltzschig HK. Hypoxia inducible factor (HIF)-1 coordinates induction of Toll-like receptors TLR2 and TLR6 during hypoxia. PLoS ONE (2007) 2:e1364. 10.1371/journal.pone.0001364 18159247 PMC2147045

[85] Tschopp J Schroder K. NLRP3 inflammasome activation: The convergence of multiple signalling pathways on ROS production? Nat Rev Immunol. (2010) 10:210–5. 10.1038/nri2725 20168318

[86] Gross O Thomas CJ Guarda G Tschopp J. The inflammasome: an integrated view. Immunol Rev. (2011) 243:136–51. 10.1111/j.1600-065X.2011.01046.x 21884173

[87] Kanneganti TD Lamkanfi M Nunez G. Intracellular NOD-like receptors in host defense and disease. Immunity (2007) 27:549–59. 10.1016/j.immuni.2007.10.002 17967410

[88] Dinarello CA. Immunological and inflammatory functions of the interleukin-1 family. Annu Rev Immunol. (2009) 27:519–50. 10.1146/annurev.immunol.021908.132612 19302047

[89] Sims JE Smith DE. The IL-1 family: regulators of immunity. Nat Rev Immunol. (2010) 10:89–102. 10.1038/nri2691 20081871

[90] Keller M Ruegg A Werner S Beer HD. Active caspase-1 is a regulator of unconventional protein secretion. Cell (2008) 132:818–31. 10.1016/j.cell.2007.12.040 18329368

[91] Latz E Xiao TS Stutz A. Activation and regulation of the inflammasomes. Nat Rev Immunol. (2013) 13:397–411. 10.1038/nri3452 23702978 PMC3807999

[92] Lamkanfi M Dixit VM. Inflammasomes and their roles in health and disease. Annu Rev Cell Dev Biol. (2012) 28:137–61. 10.1146/annurev-cellbio-101011-155745 22974247

[93] Shi J Zhao Y Wang K Shi X Wang Y Huang H. Cleavage of GSDMD by inflammatory caspases determines pyroptotic cell death. Nature (2015) 526:660–5. 10.1038/nature15514 26375003

[94] Strowig T Henao-Mejia J Elinav E Flavell R. Inflammasomes in health and disease. Nature (2012) 481:278–86. 10.1038/nature10759 22258606

[95] Benetti E Chiazza F Patel NS Collino M. The NLRP3 Inflammasome as a novel player of the intercellular crosstalk in metabolic disorders. Mediators Inflamm. (2013) 2013:678627. 10.1155/2013/678627 23843683 PMC3697790

[96] Rathinam VA Fitzgerald KA. Inflammasome complexes: emerging mechanisms and effector functions. Cell. (2016) 165:792–800. 10.1016/j.cell.2016.03.046 27153493 PMC5503689

[97] Mangan MSJ Olhava EJ Roush WR Seidel HM Glick GD Latz E Targeting the NLRP3 inflammasome in inflammatory diseases. Nat Rev Drug Discov. (2018) 17:588–606. 10.1038/nrd.2018.97 30026524

[98] Mangan MSJ Olhava EJ Roush WR Seidel HM Glick GD Latz E Targeting the NLRP3 inflammasome in inflammatory diseases. Nat Rev Drug Discov. (2018) 17:688 10.1038/nrd.2018.149 30116046

[99] Bauernfeind FG Horvath G Stutz A Alnemri ES MacDonald K Speert D. Cutting edge: NF-κB activating pattern recognition and cytokine receptors license NLRP3 inflammasome activation by regulating NLRP3 expression. J Immunol. (2009) 183:787–91. 10.4049/jimmunol.0901363 19570822 PMC2824855

[100] Franchi L Eigenbrod T Nunez G. Cutting edge: TNF-α mediates sensitization to ATP and silica via the NLRP3 inflammasome in the absence of microbial stimulation. J Immunol. (2009) 183:792–6. 10.4049/jimmunol.0900173 19542372 PMC2754237

[101] De Nardo D Latz E. NLRP3 inflammasomes link inflammation and metabolic disease. Trends Immunol. (2011) 32:373–9. 10.1016/j.it.2011.05.004 21733753 PMC3151541

[102] Dostert C Petrilli V Van Bruggen R Steele C Mossman BT Tschopp J. Innate immune activation through Nalp3 inflammasome sensing of asbestos and silica. Science (2008) 320:674–7. 10.1126/science.1156995 18403674 PMC2396588

[103] Mariathasan S Weiss DS Newton K McBride J O'Rourke K Roose-Girma M. Cryopyrin activates the inflammasome in response to toxins and ATP. Nature (2006) 440:228–32. 10.1038/nature04515 16407890

[104] Petrilli V Dostert C Muruve DA Tschopp J. The inflammasome: a danger sensing complex triggering innate immunity. Curr Opin Immunol. (2007) 19:615–22. 10.1016/j.coi.2007.09.002 17977705

[105] Haneklaus M O'Neill LA. NLRP3 at the interface of metabolism and inflammation. Immunol Rev. (2015) 265:53–62. 10.1111/imr.12285 25879283

[106] Wen H Gris D Lei Y Jha S Zhang L Huang MT. Fatty acid-induced NLRP3-ASC inflammasome activation interferes with insulin signaling. Nat Immunol. (2011) 12:408–15. 10.1038/ni.2022 21478880 PMC4090391

[107] Zhang SY Dong YQ Wang P Zhang X Yan Y Sun L. Adipocyte-derived lysophosphatidylcholine activates adipocyte and adipose tissue macrophage nod-like receptor protein 3 inflammasomes mediating homocysteine-induced insulin resistance. EBioMedicine (2018) 31:202–16. 10.1016/j.ebiom.2018.04.022 29735414 PMC6013933

[108] Tannahill GM O'Neill LA. The emerging role of metabolic regulation in the functioning of Toll-like receptors and the NOD-like receptor Nlrp3. FEBS Lett. (2011) 585:1568–72. 10.1016/j.febslet.2011.05.008 21565193

[109] Fukata M Vamadevan AS Abreu MT. Toll-like receptors (TLRs) and Nod-like receptors (NLRs) in inflammatory disorders. Semin Immunol. (2009) 21:242–53. 10.1016/j.smim.2009.06.005 19748439

[110] Fernandes-Alnemri T Kang S Anderson C Sagara J Fitzgerald KA Alnemri ES. Cutting edge: TLR signaling licenses IRAK1 for rapid activation of the NLRP3 inflammasome. J Immunol. (2013) 191:3995–9. 10.4049/jimmunol.1301681 24043892 PMC3924784

[111] Juliana C Fernandes-Alnemri T Kang S Farias A Qin F Alnemri ES. Non-transcriptional priming and deubiquitination regulate NLRP3 inflammasome activation. J Biol Chem. (2012) 287:36617–22. 10.1074/jbc.M112.407130 22948162 PMC3476327

[112] Lin KM Hu W Troutman TD Jennings M Brewer T Li X. IRAK-1 bypasses priming and directly links TLRs to rapid NLRP3 inflammasome activation. Proc Natl Acad Sci USA. (2014) 111:775–80. 10.1073/pnas.1320294111 24379360 PMC3896167

[113] Xing Y Yao X Li H Xue G Guo Q Yang G. Cutting edge: TRAF6 mediates TLR/IL-1R signaling-induced nontranscriptional priming of the NLRP3 inflammasome. J Immunol. (2017) 199:1561–6. 10.4049/jimmunol.1700175 28739881

[114] Kim SY Choi YJ Joung SM Lee BH Jung YS Lee JY. Hypoxic stress up-regulates the expression of Toll-like receptor 4 in macrophages via hypoxia-inducible factor. Immunology (2010) 129:516–24. 10.1111/j.1365-2567.2009.03203.x 20002786 PMC2842498

[115] Peyssonnaux C Cejudo-Martin P Doedens A Zinkernagel AS Johnson RS Nizet V. Cutting edge: essential role of hypoxia inducible factor-1α in development of lipopolysaccharide-induced sepsis. J Immunol. (2007) 178:7516–9. 10.4049/jimmunol.178.12.7516 17548584

[116] Hughes MM O'Neill LAJ. Metabolic regulation of NLRP3. Immunol Rev. (2018) 281:88–98. 10.1111/imr.12608 29247992

[117] Ganeshan K Chawla A. Metabolic regulation of immune responses. Annu Rev Immunol. (2014) 32:609–34. 10.1146/annurev-immunol-032713-120236 24655299 PMC5800786

[118] Zhou R Tardivel A Thorens B Choi I Tschopp J. Thioredoxin-interacting protein links oxidative stress to inflammasome activation. Nat Immunol. (2010) 11:136–40. 10.1038/ni.1831 20023662

[119] Horng T Hotamisligil GS. Linking the inflammasome to obesity-related disease. Nat Med. (2011) 17:164–5. 10.1038/nm0211-164 21297609

[120] Stienstra R Joosten LA Koenen T van Tits B van Diepen JA van den Berg SA. The inflammasome-mediated caspase-1 activation controls adipocyte differentiation and insulin sensitivity. Cell Metab. (2010) 12:593–605. 10.1016/j.cmet.2010.11.011 21109192 PMC3683568

[121] Stienstra R van Diepen JA Tack CJ Zaki MH van de Veerdonk FL Perera D. Inflammasome is a central player in the induction of obesity and insulin resistance. Proc Natl Acad Sci USA. (2011) 108:15324–9. 10.1073/pnas.1100255108 21876127 PMC3174591

[122] Vandanmagsar B Youm YH Ravussin A Galgani JE Stadler K Mynatt RL. The NLRP3 inflammasome instigates obesity-induced inflammation and insulin resistance. Nat Med. (2011) 17:179–88. 10.1038/nm.2279 21217695 PMC3076025

[123] Lamkanfi M Mueller JL Vitari AC Misaghi S Fedorova A Deshayes K. Glyburide inhibits the Cryopyrin/Nalp3 inflammasome. J Cell Biol. (2009) 187:61–70. 10.1083/jcb.200903124 19805629 PMC2762099

[124] Masters SL Dunne A Subramanian SL Hull RL Tannahill GM Sharp FA. Activation of the NLRP3 inflammasome by islet amyloid polypeptide provides a mechanism for enhanced IL-1β in type 2 diabetes. Nat Immunol. (2010) 11:897–904. 10.1038/ni.1935 20835230 PMC3103663

[125] Wen H Ting JP O'Neill LA. A role for the NLRP3 inflammasome in metabolic diseases–did Warburg miss inflammation? Nat Immunol. (2012) 13:352–7. 10.1038/ni.2228 22430788 PMC4090390

[126] Netea MG Joosten LA Lewis E Jensen DR Voshol PJ Kullberg BJ. Deficiency of interleukin-18 in mice leads to hyperphagia, obesity and insulin resistance. Nat Med. (2006) 12:650–6. 10.1038/nm1415 16732281

[127] Esposito K Pontillo A Ciotola M Di Palo C Grella E Nicoletti G. Weight loss reduces interleukin-18 levels in obese women. J Clin Endocrinol Metab. (2002) 87:3864–6. 10.1210/jcem.87.8.8781 12161523

[128] Hung J McQuillan BM Chapman CM Thompson PL Beilby JP. Elevated interleukin-18 levels are associated with the metabolic syndrome independent of obesity and insulin resistance. Arterioscler Thromb Vasc Biol. (2005) 25:1268–73. 10.1161/01.ATV.0000163843.70369.12 15790931

[129] Murphy AJ Kraakman MJ Kammoun HL Dragoljevic D Lee MK Lawlor KE. IL-18 production from the NLRP1 inflammasome prevents obesity and metabolic syndrome. Cell Metab. (2016) 23:155–64. 10.1016/j.cmet.2015.09.024 26603191

[130] Stienstra R Tack CJ Kanneganti TD Joosten LA Netea MG. The inflammasome puts obesity in the danger zone. Cell Metab. (2012) 15:10–8. 10.1016/j.cmet.2011.10.011 22225872

[131] Tack CJ Stienstra R Joosten LA Netea MG. Inflammation links excess fat to insulin resistance: the role of the interleukin-1 family. Immunol Rev. (2012) 249:239–52. 10.1111/j.1600-065X.2012.01145.x 22889226

[132] Maloy KJ Powrie F. Intestinal homeostasis and its breakdown in inflammatory bowel disease. Nature (2011) 474:298–306. 10.1038/nature10208 21677746

[133] Allen IC TeKippe EM Woodford RM Uronis JM Holl EK Rogers AB. The NLRP3 inflammasome functions as a negative regulator of tumorigenesis during colitis-associated cancer. J Exp Med. (2010) 207:1045–56. 10.1084/jem.20100050 20385749 PMC2867287

[134] Bauer C Duewell P Mayer C Lehr HA Fitzgerald KA Dauer M. Colitis induced in mice with dextran sulfate sodium (DSS) is mediated by the NLRP3 inflammasome. Gut (2010) 59:1192–9. 10.1136/gut.2009.197822 20442201

[135] Hirota SA Ng J Lueng A Khajah M Parhar K Li Y. NLRP3 inflammasome plays a key role in the regulation of intestinal homeostasis. Inflamm Bowel Dis. (2011) 17:1359–72. 10.1002/ibd.21478 20872834 PMC3026862

[136] Zaki MH Boyd KL Vogel P Kastan MB Lamkanfi M Kanneganti TD. The NLRP3 inflammasome protects against loss of epithelial integrity and mortality during experimental colitis. Immunity (2010) 32:379–91. 10.1016/j.immuni.2010.03.003 20303296 PMC2982187

[137] Dinarello CA. The IL-1 family and inflammatory diseases. Clin Exp Rheumatol. (2002) 20:S1–13. 14989423

[138] Siegmund B Lehr HA Fantuzzi G Dinarello CA IL-1β -converting enzyme (caspase-1) in intestinal inflammation. Proc Natl Acad Sci USA. (2001) 98:13249–54. 10.1073/pnas.231473998 11606779 PMC60856

[139] Drexler SK Yazdi AS. Complex roles of inflammasomes in carcinogenesis. Cancer J. (2013) 19:468–72. 10.1097/PPO.0000000000000004 24270345

[140] Lissner D Siegmund B. The multifaceted role of the inflammasome in inflammatory bowel diseases. Sci World J. (2011) 11:1536–47. 10.1100/tsw.2011.139 21805022 PMC5596529

[141] Huber S Gagliani N Zenewicz LA Huber FJ Bosurgi L Hu B. IL-22BP is regulated by the inflammasome and modulates tumorigenesis in the intestine. Nature (2012) 491:259–63. 10.1038/nature11535 23075849 PMC3493690

[142] Zaki MH Lamkanfi M Kanneganti TD. The Nlrp3 inflammasome: contributions to intestinal homeostasis. Trends Immunol. (2011) 32:171–9. 10.1016/j.it.2011.02.002 21388882 PMC3070791

[143] Wang H Wang Y Du Q Lu P Fan H Lu J. Inflammasome-independent NLRP3 is required for epithelial-mesenchymal transition in colon cancer cells. Exp Cell Res. (2016) 342:184–92. 10.1016/j.yexcr.2016.03.009 26968633

[144] Du Q Wang Q Fan H Wang J Liu X Wang H. Dietary cholesterol promotes AOM-induced colorectal cancer through activating the NLRP3 inflammasome. Biochem Pharmacol. (2016) 105:42–54. 10.1016/j.bcp.2016.02.017 26921636

[145] Guo W Sun Y Liu W Wu X Guo L Cai P. Small molecule-driven mitophagy-mediated NLRP3 inflammasome inhibition is responsible for the prevention of colitis-associated cancer. Autophagy (2014) 10:972–85. 10.4161/auto.28374 24879148 PMC4091180

[146] Villani AC Lemire M Fortin G Louis E Silverberg MS Collette C. Common variants in the NLRP3 region contribute to Crohn's disease susceptibility. Nat Genet. (2009) 41:71–6. 10.1038/ng.285 19098911 PMC2728932

[147] Ungerback J Belenki D Jawad ul-Hassan A Fredrikson M Fransen K Elander N. Genetic variation and alterations of genes involved in NFκB/TNFAIP3- and NLRP3-inflammasome signaling affect susceptibility and outcome of colorectal cancer. Carcinogenesis (2012) 33:2126–34. 10.1093/carcin/bgs256 22843550

[148] Voronov E Apte RN. IL-1 in colon inflammation, colon carcinogenesis and invasiveness of colon cancer. Cancer Microenviron. (2015) 8:187–200. 10.1007/s12307-015-0177-7 26686225 PMC4715003

[149] Krelin Y Voronov E Dotan S Elkabets M Reich E Fogel M. Interleukin-1β-driven inflammation promotes the development and invasiveness of chemical carcinogen-induced tumors. Cancer Res. (2007) 67:1062–71. 10.1158/0008-5472.CAN-06-2956 17283139

[150] Miki C Konishi N Ojima E Hatada T Inoue Y Kusunoki M. C-reactive protein as a prognostic variable that reflects uncontrolled up-regulation of the IL-1-IL-6 network system in colorectal carcinoma. Dig Dis Sci. (2004) 49:970–6. 10.1023/B:DDAS.0000034556.48527.6e 15309885

[151] Tu S Bhagat G Cui G Takaishi S Kurt-Jones EA Rickman B. Overexpression of interleukin-1β induces gastric inflammation and cancer and mobilizes myeloid-derived suppressor cells in mice. Cancer Cell (2008) 14:408–19. 10.1016/j.ccr.2008.10.011 18977329 PMC2586894

[152] Voronov E Shouval DS Krelin Y Cagnano E Benharroch D Iwakura Y. IL-1 is required for tumor invasiveness and angiogenesis. Proc Natl Acad Sci USA. (2003) 100:2645–50. 10.1073/pnas.0437939100 12598651 PMC151394

[153] Apte RN Voronov E. Interleukin-1–a major pleiotropic cytokine in tumor-host interactions. Semin Cancer Biol. (2002) 12:277–90. 10.1016/S1044-579X(02)00014-7 12147202

[154] Lin WW Karin M. A cytokine-mediated link between innate immunity, inflammation, and cancer. J Clin Invest. (2007) 117:1175–83. 10.1172/JCI31537 17476347 PMC1857251

[155] Lopetuso LR Chowdhry S Pizarro TT. Opposing functions of classic and novel IL-1 family members in gut health and disease. Front Immunol. (2013) 4:181. 10.3389/fimmu.2013.00181 23847622 PMC3705591

[156] Mahida YR Wu K Jewell DP. Enhanced production of interleukin 1-β by mononuclear cells isolated from mucosa with active ulcerative colitis of Crohn's disease. Gut (1989) 30:835–8. 10.1136/gut.30.6.835 2787769 PMC1434123

[157] Stronati L Negroni A Pierdomenico M D'Ottavio C Tirindelli D Di Nardo G. Altered expression of innate immunity genes in different intestinal sites of children with ulcerative colitis. Dig Liver Dis. (2010) 42:848–53. 10.1016/j.dld.2010.04.003 20452301

[158] Li Y Wang L Pappan L Galliher-Beckley A Shi J. IL-1β promotes stemness and invasiveness of colon cancer cells through Zeb1 activation. Mol Cancer (2012) 11:87. 10.1186/1476-4598-11-87 23174018 PMC3532073

[159] Wang L Liu Z Li Y Pappan L Galliher-Beckley A Shi J. Pro-inflammatory cytokine interleukin-1β promotes the development of intestinal stem cells. Inflamm Res. (2012) 61:1085–92. 10.1007/s00011-012-0501-3 22706317

[160] He C Yu T Shi Y Ma C Yang W Fang L. MicroRNA 301A promotes intestinal inflammation and colitis-associated cancer development by inhibiting BTG1. Gastroenterology (2017) 152:1434–48.e15. 10.1053/j.gastro.2017.01.049 28193514

[161] Choi S Chung H Hong H Kim SY Kim SE Seoh JY. Inflammatory hypoxia induces syndecan-2 expression through IL-1β-mediated FOXO3a activation in colonic epithelia. FASEB J. (2017) 31:1516–30. 10.1096/fj.201601098R 28031321

[162] Gunter MJ Canzian F Landi S Chanock SJ Sinha R Rothman N. Inflammation-related gene polymorphisms and colorectal adenoma. Cancer Epidemiol Biomarkers Prev. (2006) 15:1126–31. 10.1158/1055-9965.EPI-06-0042 16775170

[163] Maeda S Hsu LC Liu H Bankston LA Iimura M Kagnoff MF. Nod2 mutation in Crohn's disease potentiates NF-κB activity and IL-1β processing. Science (2005) 307:734–8. 10.1126/science.1103685 15692052

[164] Jedinak A Dudhgaonkar S Sliva D. Activated macrophages induce metastatic behavior of colon cancer cells. Immunobiology (2010) 215:242–9. 10.1016/j.imbio.2009.03.004 19457576

[165] Kimura YN Watari K Fotovati A Hosoi F Yasumoto K Izumi H. Inflammatory stimuli from macrophages and cancer cells synergistically promote tumor growth and angiogenesis. Cancer Sci. (2007) 98:2009–18. 10.1111/j.1349-7006.2007.00633.x 17924976 PMC11159678

[166] Pelegrin P Barroso-Gutierrez C Surprenant A. P2X7 receptor differentially couples to distinct release pathways for IL-1β in mouse macrophage. J Immunol. (2008) 180:7147–57. 10.4049/jimmunol.180.11.7147 18490713

[167] Kaler P Augenlicht L Klampfer L. Macrophage-derived IL-1β stimulates Wnt signaling and growth of colon cancer cells: a crosstalk interrupted by vitamin D3. Oncogene (2009) 28:3892–902. 10.1038/onc.2009.247 19701245 PMC2783659

[168] Kaler P Galea V Augenlicht L Klampfer L. Tumor associated macrophages protect colon cancer cells from TRAIL-induced apoptosis through IL-1β-dependent stabilization of Snail in tumor cells. PLoS ONE (2010) 5:e11700. 10.1371/journal.pone.0011700 20661477 PMC2908545

[169] Kaler P Godasi BN Augenlicht L Klampfer L. The NF-κB/AKT-dependent induction of Wnt signaling in colon cancer cells by macrophages and IL-1β. Cancer Microenviron. (2009) 2:69–80. 10.1007/s12307-009-0030-y 19779850 PMC2787930

[170] Arendt LM McCready J Keller PJ Baker DD Naber SP Seewaldt V. Obesity promotes breast cancer by CCL2-mediated macrophage recruitment and angiogenesis. Cancer Res. (2013) 73:6080–93. 10.1158/0008-5472.CAN-13-0926 23959857 PMC3824388

[171] Nakao S Kuwano T Tsutsumi-Miyahara C Ueda S Kimura YN Hamano S. Infiltration of COX-2-expressing macrophages is a prerequisite for IL-1 β-induced neovascularization and tumor growth. J Clin Invest. (2005) 115:2979–91. 10.1172/JCI23298 16239969 PMC1257532

[172] Dinarello CA Novick D Puren AJ Fantuzzi G Shapiro L Muhl H. Overview of interleukin-18: more than an interferon-γ inducing factor. J Leukoc Biol. (1998) 63:658–64. 10.1002/jlb.63.6.658 9620656

[173] Okamura H Tsutsi H Komatsu T Yutsudo M Hakura A Tanimoto T. Cloning of a new cytokine that induces IFN-γ production by T cells. Nature (1995) 378:88–91. 10.1038/378088a0 7477296

[174] Komai-Koma M Gracie JA Wei XQ Xu D Thomson N McInnes IB. Chemoattraction of human T cells by IL-18. J Immunol. (2003) 170:1084–90. 10.4049/jimmunol.170.2.1084 12517977

[175] Pizarro TT Michie MH Bentz M Woraratanadharm J Smith MF Jr Foley E. IL-18, a novel immunoregulatory cytokine, is up-regulated in Crohn's disease: expression and localization in intestinal mucosal cells. J Immunol. (1999) 162:6829–35. 10352304

[176] Ye ZB Ma T Li H Jin XL Xu HM. Expression and significance of intratumoral interleukin-12 and interleukin-18 in human gastric carcinoma. World J Gastroenterol. (2007) 13:1747–51. 10.3748/wjg.v13.i11.1747 17461482 PMC4146958

[177] Salcedo R Worschech A Cardone M Jones Y Gyulai Z Dai RM. MyD88-mediated signaling prevents development of adenocarcinomas of the colon: role of interleukin 18. J Exp Med. (2010) 207:1625–36. 10.1084/jem.20100199 20624890 PMC2916129

[178] Kim KE Song H Kim TS Yoon D Kim CW Bang SI. Interleukin-18 is a critical factor for vascular endothelial growth factor-enhanced migration in human gastric cancer cell lines. Oncogene (2007) 26:1468–76. 10.1038/sj.onc.1209926 17001321

[179] Nowarski R Jackson R Gagliani N de Zoete MR Palm NW Bailis W. Epithelial IL-18 equilibrium controls barrier function in colitis. Cell (2015) 163:1444–56. 10.1016/j.cell.2015.10.072 26638073 PMC4943028

[180] Ahmad R Al-Mass A Al-Ghawas D Shareif N Zghoul N Melhem M. Interaction of osteopontin with IL-18 in obese individuals: implications for insulin resistance. PLoS ONE (2013) 8:e63944. 10.1371/journal.pone.0063944 23675517 PMC3652828

[181] Esposito K Nappo F Giugliano F Di Palo C Ciotola M Barbieri M. Cytokine milieu tends toward inflammation in type 2 diabetes. Diabetes Care (2003) 26:1647. 10.2337/diacare.26.5.1647 12716849

[182] Bruun JM Stallknecht B Helge JW Richelsen B. Interleukin-18 in plasma and adipose tissue: effects of obesity, insulin resistance, and weight loss. Eur J Endocrinol. (2007) 157:465–71. 10.1530/EJE-07-0206 17893261

[183] Wood IS Wang B Jenkins JR Trayhurn P The pro-inflammatory cytokine IL-18 is expressed in human adipose tissue and strongly upregulated by TNF-α in human adipocytes. Biochem Biophys Res Commun. (2005) 337:422–9. 10.1016/j.bbrc.2005.09.068 16188228

[184] Skurk T Kolb H Muller-Scholze S Rohrig K Hauner H Herder C. The proatherogenic cytokine interleukin-18 is secreted by human adipocytes. Eur J Endocrinol. (2005) 152:863–8. 10.1530/eje.1.01897 15941925

[185] Liu Z Brooks RS Ciappio ED Kim SJ Crott JW Bennett G. Diet-induced obesity elevates colonic TNF-α in mice and is accompanied by an activation of Wnt signaling: a mechanism for obesity-associated colorectal cancer. J Nutr Biochem. (2012) 23:1207–13. 10.1016/j.jnutbio.2011.07.002 22209007 PMC4142203

[186] Williams SC. Link between obesity and cancer. Proc Natl Acad Sci USA. (2013) 110:8753–4. 10.1073/pnas.1308182110 23716575 PMC3670342

[187] Kolb R Phan L Borcherding N Liu Y Yuan F Janowski AM. Obesity-associated NLRC4 inflammasome activation drives breast cancer progression. Nat Commun. (2016) 7:13007. 10.1038/ncomms13007 27708283 PMC5059727

[188] Hillenbrand A Fassler J Huber N Xu P Henne-Bruns D Templin M. Changed adipocytokine concentrations in colorectal tumor patients and morbidly obese patients compared to healthy controls. BMC Cancer (2012) 12:545. 10.1186/1471-2407-12-545 23173608 PMC3523089

[189] Bing C. Is interleukin-1β a culprit in macrophage-adipocyte crosstalk in obesity? Adipocyte (2015) 4:149–52. 10.4161/21623945.2014.979661 26167419 PMC4496963

[190] Correa LH Correa R Farinasso CM de Sant'Ana Dourado LP Magalhaes KG. Adipocytes and macrophages interplay in the orchestration of tumor microenvironment: new implications in cancer progression. Front Immunol. (2017) 8:1129. 10.3389/fimmu.2017.01129 28970834 PMC5609576

[191] Weichand B Popp R Dziumbla S Mora J Strack E Elwakeel E. S1PR1 on tumor-associated macrophages promotes lymphangiogenesis and metastasis via NLRP3/IL-1β. J Exp Med. (2017) 214:2695–713. 10.1084/jem.20160392 28739604 PMC5584110

[192] Kolb R Liu GH Janowski AM Sutterwala FS Zhang W. Inflammasomes in cancer: a double-edged sword. Protein Cell (2014) 5:12–20. 10.1007/s13238-013-0001-4 24474192 PMC3938856

[193] Gómez-Ambrosi J Silva C Catalán V Rodríguez A Galofre JC Escalada J. Clinical usefulness of a new equation for estimating body fat. Diabetes Care (2012) 35:383–8. 10.2337/dc11-1334 22179957 PMC3263863

[194] Frühbeck G Gómez-Ambrosi J. Control of body weight: a physiologic and transgenic perspective. Diabetologia (2003) 46:143–72. 10.1007/s00125-003-1053-4 12627314

[195] Poulain-Godefroy O Lecoeur C Pattou F Frühbeck G Froguel P. Inflammation is associated with a decrease of lipogenic factors in omental fat in women. Am J Physiol Regul Integr Comp Physiol. (2008) 295:R1–7. 10.1152/ajpregu.00926.2007 18448614

[196] Catalán V Gómez-Ambrosi J Rotellar F Silva C Rodríguez A Salvador J. Validation of endogenous control genes in human adipose tissue: relevance to obesity and obesity-associated type 2 diabetes mellitus. Horm Metab Res. (2007) 39:495–500. 10.1055/s-2007-982502 17611901

[197] Moreno-Navarrete JM Ortega F Serino M Luche E Waget A Pardo G. Circulating lipopolysaccharide-binding protein (LBP) as a marker of obesity-related insulin resistance. Int J Obes. (2012) 36:1442–9. 10.1038/ijo.2011.256 22184060

[198] Frühbeck G Gómez-Ambrosi J. Modulation of the leptin-induced white adipose tissue lipolysis by nitric oxide. Cell Signal. (2001) 13:827–33. 10.1016/S0898-6568(01)00211-X 11583918

[199] Frühbeck G Gómez-Ambrosi J Salvador J. Leptin-induced lipolysis opposes the tonic inhibition of endogenous adenosine in white adipocytes. FASEB J. (2001) 15:333–40. 10.1096/fj.00-0249com 11156949

[200] Ortega FJ Mayas D Moreno-Navarrete JM Catalán V Gómez-Ambrosi J Esteve E. The gene expression of the main lipogenic enzymes is downregulated in visceral adipose tissue of obese subjects. Obesity (2010) 18:13–20. 10.1038/oby.2009.202 19543203

[201] Frühbeck G. Obesity: aquaporin enters the picture. Nature (2005) 438:436–7. 10.1038/438436b 16306977

[202] Frühbeck G Gómez-Ambrosi J. Rationale for the existence of additional adipostatic hormones. FASEB J. (2001) 15:1996–2006. 10.1096/fj.00-0829hyp 11532980

[203] Gómez-Ambrosi J Salvador J Paramo JA Orbe J de Irala J Díez-Caballero A. Involvement of leptin in the association between percentage of body fat and cardiovascular risk factors. Clin Biochem. (2002) 35:315–20. 10.1016/S0009-9120(02)00320-X 12135695

[204] Sabater M Moreno-Navarrete JM Ortega FJ Pardo G Salvador J Ricart W. Circulating pigment epithelium-derived factor levels are associated with insulin resistance and decrease after weight loss. J Clin Endocrinol Metab. (2010) 95:4720–8. 10.1210/jc.2010-0630 20631025

[205] Pardo M Crujeiras AB Amil M Aguera Z Jimenez-Murcia S Banos R. Association of irisin with fat mass, resting energy expenditure, and daily activity in conditions of extreme body mass index. Int J Endocrinol. (2014) 2014:857270. 10.1155/2014/857270 24864142 PMC4016898

[206] Dagenais M Saleh M. Linking cancer-induced Nlrp3 inflammasome activation to efficient NK cell-mediated immunosurveillance. Oncoimmunology (2016) 5:e1129484. 10.1080/2162402X.2015.1129484 27467946 PMC4910718

[207] Saleh M Trinchieri G. Innate immune mechanisms of colitis and colitis-associated colorectal cancer. Nat Rev Immunol. (2011) 11:9–20. 10.1038/nri2891 21151034

[208] Ghiringhelli F Apetoh L Tesniere A Aymeric L Ma Y Ortiz C. Activation of the NLRP3 inflammasome in dendritic cells induces IL-1β -dependent adaptive immunity against tumors. Nat Med. (2009) 15:1170–8. 10.1038/nm.2028 19767732

[209] Garaude J Kent A van Rooijen N Blander JM. Simultaneous targeting of toll- and nod-like receptors induces effective tumor-specific immune responses. Sci Transl Med. (2012) 4:120ra16. 10.1126/scitranslmed.3002868 22323829

[210] Dietsch GN Lu H Yang Y Morishima C Chow LQ Disis ML Coordinated activation of toll-like receptor 8 (TLR8) and NLRP3 by the TLR8 agonist, VTX-2337, ignites tumoricidal natural killer cell activity. PLoS ONE (2016) 11:e0148764 10.1371/journal.pone.0148764 26928328 PMC4771163

[211] Dinarello CA Simon A van der Meer JW. Treating inflammation by blocking interleukin-1 in a broad spectrum of diseases. Nat Rev Drug Discov. (2012) 11:633–52. 10.1038/nrd3800 22850787 PMC3644509

[212] Jiang H He H Chen Y Huang W Cheng J Ye J. Identification of a selective and direct NLRP3 inhibitor to treat inflammatory disorders. J Exp Med. (2017) 214:3219–38. 10.1084/jem.20171419 29021150 PMC5679172

[213] Chiazza F Couturier-Maillard A Benetti E Mastrocola R Nigro D Cutrin JC. Targeting the NLRP3 inflammasome to reduce diet-induced metabolic abnormalities in mice. Mol Med. (2015) 21:1025–37. 10.2119/molmed.2015.00104 26623925 PMC4982477

[214] Youm YH Nguyen KY Grant RW Goldberg EL Bodogai M Kim D. The ketone metabolite β-hydroxybutyrate blocks NLRP3 inflammasome-mediated inflammatory disease. Nat Med. (2015) 21:263–9. 10.1038/nm.3804 25686106 PMC4352123

[215] Frühbeck G. Bariatric and metabolic surgery: a shift in eligibility and success criteria. Nat Rev Endocrinol. (2015) 11:465–77. 10.1038/nrendo.2015.84 26055046

[216] Frühbeck G Kiortsis DN Catalán V. Precision medicine: diagnosis and management of obesity. Lancet Diabetes Endocrinol. (2018) 6:164–6. 10.1016/S2213-8587(17)30312-1 28919063

